# The Dysferlin Domain-Only Protein, Spo73, Is Required for Prospore Membrane Extension in *Saccharomyces cerevisiae*

**DOI:** 10.1128/mSphere.00038-15

**Published:** 2015-12-16

**Authors:** Yuuya Okumura, Tsuyoshi S. Nakamura, Takayuki Tanaka, Ichiro Inoue, Yasuyuki Suda, Tetsuo Takahashi, Hideki Nakanishi, Shugo Nakamura, Xiao-Dong Gao, Hiroyuki Tachikawa

**Affiliations:** a Department of Applied Biological Chemistry, Graduate School of Agricultural and Life Sciences, The University of Tokyo, Bunkyo-ku, Tokyo, Japan; b Department of Molecular Cell Biology, Graduate School of Comprehensive Human Sciences and Institute of Basic Medical Sciences, University of Tsukuba, Tsukuba, Japan; c Laboratory of Glycobiology and Glycotechnology, Department of Applied Biochemistry, School of Engineering, Tokai University, Hiratsuka City, Kanagawa, Japan; d Key Laboratory of Carbohydrate Chemistry and Biotechnology, Ministry of Education, School of Biotechnology, Jiangnan University, Wuxi, China; e Department of Biotechnology, Graduate School of Agricultural and Life Sciences, The University of Tokyo, Bunkyo-ku, Tokyo, Japan; Carnegie Mellon University

**Keywords:** *Saccharomyces cerevisiae*, sporulation, prospore membrane, dysferlin domain

## Abstract

Sporulation of *Saccharomyces cerevisiae* is a developmental process in which an ascus containing four haploid spores forms from a diploid cell. During this process, newly formed membrane structures called prospore membranes extend along the nuclear envelope and engulf and package daughter nuclei along with cytosol and organelles to form precursors of spores. Proteins involved in prospore membrane extension, Vps13 and Spo71, have recently been reported; however, the overall mechanism of membrane extension remains unclear. Here, we identified Spo73 as an additional factor involved in prospore membrane extension. Analysis of a *spo73∆* mutant revealed that it shows defects similar to those of a *spo71∆* mutant during prospore membrane formation. Spo73 localizes to the prospore membrane, and this localization is independent of Spo71 and Vps13. In contrast, a Spo73 protein carrying mutations in a surface basic patch mislocalizes to the cytoplasm and overexpression of Spo71 can partially rescue localization to the prospore membrane. Similar to *spo71∆* mutants, *spo73∆* mutants display genetic interactions with the mutations in the *SMA2* and *SPO1* genes involved in prospore membrane bending. Further, our bioinformatic analysis revealed that Spo73 is a dysferlin domain-only protein. Thus, these results suggest that a dysferlin domain-only protein, Spo73, functions with a dual pleckstrin homology domain protein, Spo71, in prospore membrane extension. Analysis of Spo73 will provide insights into the conserved function of dysferlin domains, which is related to dysferlinopathy.

**IMPORTANCE** Prospore membrane formation consists of *de novo* double-membrane formation, which occurs during the developmental process of sporulation in *Saccharomyces cerevisiae*. Membranes are formed into their proper size and shape, and thus, prospore membrane formation has been studied as a general model of membrane formation. We identified *SPO73*, previously shown to be required for spore wall formation, as an additional gene involved in prospore membrane extension. Genetic and cell biological analyses suggested that Spo73 functions on the prospore membrane with other factors in prospore membrane extension, counteracting the bending force of the prospore membrane. Spo73 is the first dysferlin domain-only protein ever analyzed. The dysferlin domain is conserved from yeast to mammals and is found in dysferlin proteins, which are involved in dysferlinopathy, although the precise function of the domain is unknown. Continued analysis of Spo73 will contribute to our understanding of the function of dysferlin domains and dysferlinopathy.

## Abstract (precis)

Prospore membrane formation consists of *de novo* double-membrane formation, which occurs during the developmental process of sporulation in *Saccharomyces cerevisiae*. Membranes are formed into their proper size and shape, and thus, prospore membrane formation has been studied as a general model of membrane formation. We identified *SPO73*, previously shown to be required for spore wall formation, as an additional gene involved in prospore membrane extension. Genetic and cell biological analyses suggested that Spo73 functions on the prospore membrane with other factors in prospore membrane extension, counteracting the bending force of the prospore membrane. Spo73 is the first dysferlin domain-only protein ever analyzed. The dysferlin domain is conserved from yeast to mammals and is found in dysferlin proteins, which are involved in dysferlinopathy, although the precise function of the domain is unknown. Continued analysis of Spo73 will contribute to our understanding of the function of dysferlin domains and dysferlinopathy.

## INTRODUCTION

The yeast *Saccharomyces cerevisiae* initiates the developmental process of sporulation under conditions of nitrogen deprivation and the presence of a nonfermentable carbon source. Four spores are formed inside a diploid cell, which turns into an ascus. In this process, four new membranes called prospore membranes are formed in the cytosol of the diploid cell during meiosis II, encapsulate the four nuclei, respectively, and become plasma membranes of each spore (1–3).

**FIG 1:**
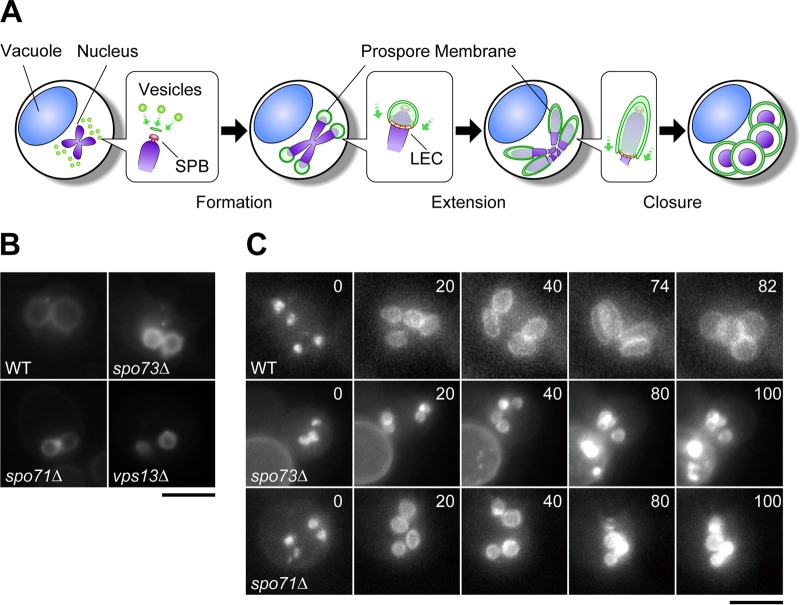
Observation of prospore membranes in wild-type (WT) and *spo73∆* and *spo71∆* mutant cells. (A) Overview of the stages of prospore membrane formation. SPB, spindle pole body. (B) Wild-type and *vps13∆*, *spo73∆*, and *spo71∆* mutant cells in the BY4743 background expressing *GFP-SPO20^51-91^* were sporulated for 24 to 36 h and subjected to fluorescence microscopy. (C) AN120 (wild-type), TC545 (*spo73∆* mutant), and TC581 (*spo71∆* mutant) cells expressing *GFP-SPO20^51-91^* were sporulated for 6.5 h and subjected to time-lapse microscopic analysis. The time after the start of observation is shown in minutes. Scale bars, 5 μm.

A prospore membrane grows through a series of discrete morphological stages (Fig. 1A). (i) It appears as a small horseshoe-shaped structure at the cytoplasmic surface of the spindle pole body by fusion of post-Golgi vesicles; (ii) it expands into a small round shape; (iii) as meiosis II progresses, it extends along the nuclear envelope and is shaped into a tube-like or cashew nut-like structure engulfing the nucleus, cytoplasm, and organelles; and (iv) at the end of the meiotic process, it quickly turns into a round shape of mature size, just following or at the same time as the closure of its leading edge (4–6). Prospore membrane formation is a process of *de novo* membrane formation into a proper size and shape; thus, it is considered to be a model of biological membrane formation.

Previous studies have shown that *VPS13* and *SPO71* function in prospore membrane extension. *VPS13* was originally identified as a gene involved in *trans*-Golgi network–endosomal cycling of a subset of membrane proteins and was shown to be required for vacuolar protein sorting (7). *VPS13* also has a function in sporulation; *vps13∆* mutant cells can only form small prospore membranes and are sporulation defective (8, 9). *SPO71*, which encodes a dual pleckstrin homology (PH) domain protein, was identified in screening for genes upregulated during and required for sporulation (10). *spo71∆* mutant cells also form small prospore membranes, although the defect looks less severe than that in *vps13∆* mutant cells (11). An analysis of the relationship between these proteins revealed that Spo71 interacts with Vps13 and recruits it to the prospore membrane, where both proteins colocalize (12). Further, Vps13 and Spo71 are suggested to have a function in regulating the levels of phosphatidylinositol 4-phosphate (PI4P) in prospore membranes; PI4P pools of prospore membranes are reduced in *vps13∆* and *spo71∆* mutants (12). PI4P has a variety of cellular roles, including the regulation of membrane traffic through the Golgi apparatus (13). Thus, decreased levels of PI4P in the prospore membrane of these mutant cells could cause some defect in prospore membrane extension. However, the overall mechanism of prospore membrane extension is still unknown.

The spore membrane-bending pathway and the leading-edge complex (LEC), both of which are important for the shape of the prospore membrane, could also contribute to proper prospore membrane extension. The spore membrane-bending pathway consists of a phospholipase B, Spo1; a membrane protein, Sma2; and glycophosphatidylinositol-anchored proteins, including Spo19 (14). These proteins are suggested to exert a bending force toward the nucleus during prospore membrane growth to facilitate its proper shaping; in *spo1∆* and *sma2∆* mutants, prospore membranes become wide and open. The LEC is a protein complex that localizes to the lip of each prospore membrane and consists of at least four proteins, including Don1 and an essential component, Ssp1 (15–17). Deletion of the *SSP1* gene causes the formation of unnaturally elongated prospore membranes that stick tightly to the nuclear envelope (15), suggesting that the LEC counteracts the bending force of the prospore membrane until it is removed at the onset of closure of the prospore membrane (4, 18).

In this study, we performed a screening for additional genes involved in prospore membrane extension and identified *SPO73*, which was previously suggested to be involved in spore wall formation (19). *spo73∆* mutant cells show phenotypes similar to those of *spo71∆* mutant cells. We also show that a Spo73 protein carrying mutations in a surface basic patch mislocalizes to the cytoplasm and overexpression of Spo71 can partially rescue localization to the prospore membrane. Additionally, we find that, as is the case with *SPO71* (11), *SPO73* genetically interacts with *SPO1* and *SMA2*; double mutants partially recover sporulation, suggesting that they have opposing functions in prospore membrane extension. Further, our bioinformatic analysis revealed that Spo73 is a dysferlin domain-only protein. Dysferlin domains are found in a subset of ferlin family proteins, including dysferlin, which is associated with the plasma membrane, t-tubule network, and intracellular vesicles in skeletal muscle and is involved in Ca^2+^-dependent plasma membrane repair (20). Mutations in this domain of the dysferlin protein cause dysferlinopathies, including limb girdle muscular dystrophy type 2B and Miyoshi myopathy (20–23), although the precise function of the domain is not known. Thus, analysis of Spo73 will help our understanding of the function of dysferlin domains and may contribute to understanding of the molecular basis of dysferlinopathies.

## RESULTS

### Screening for mutants defective in prospore membrane extension identified the *spo73∆* mutant.

Previous screening for mutants defective in prospore membrane formation identified eight mutants (8). One of them, the *vps13∆* mutant, formed small round prospore membranes, indicating that *VPS13* is required for proper prospore membrane extension. However, the overall mechanism of prospore membrane extension was almost unknown. To obtain additional mutants defective in prospore membrane extension, we expanded the screening to 293 mutants (for a list of corresponding genes, see Table S1 in the supplemental material) that were selected from the deletion collection based on previous reports of genome-wide analyses of sporulation defects (24–26). Mutants analyzed in previous screenings, mutants with changes in functionally characterized genes, and strains that did not grow well were excluded from this study, and the *vps13∆* mutant was used as a control. A green fluorescent protein (GFP)-tagged Spo20 lipid-binding domain (27) was expressed as a prospore membrane marker in those mutants and observed under a microscope during sporulation, one by one. We identified two mutants, the *spo71∆* and *spo73∆* mutants, that showed only a small prospore membrane pattern suggestive of a prospore membrane extension defect (Fig. 1B). During the course of our study, Spo71 was reported to be involved in prospore membrane extension (11). Therefore, we focused on Spo73. *SPO73* encodes a 143-amino-acid protein with a dysferlin C-terminal domain in its C-terminal region (19). Analysis with HHpred revealed that the Spo73 protein also contains a dysferlin N-terminal domain in its N-terminal region; thus, Spo73 is a dysferlin domain-only protein. As all of the dysferlin domain-containing proteins characterized so far contain other domains (20, 28), such as transmembrane and/or c2 domains, Spo73 is the first example of a dysferlin domain-only protein. Spo73 was first identified in a genome-wide screening for genes upregulated during and involved in sporulation and has been shown to be required for spore wall formation (10, 19). No relatedness of Spo73 to prospore membrane formation has previously been reported.

10.1128/mSphere.00038-15.1 Table S1 Genes screened in this study. Download Table S1, PDF file, 0.08 MB. Copyright © 2015 Okumura et al. 2015 Okumura et al. This content is distributed under the terms of the Creative Commons Attribution 4.0 International license.

The small prospore membrane phenotype of the *spo73∆* mutant was confirmed by time-lapse video microscopy. To observe sporulation more precisely, fast-sporulating SK1 background strains were used. In wild-type cells, prospore membranes appeared as four horseshoe-shaped membrane structures, enlarged to form four small round shapes, extended to form tube-like shapes, and finally closed and quickly attained their spherical shapes (Fig. 1C; see Movie S1 in the supplemental material). In *spo73∆* mutant cells, prospore membranes appeared and enlarged to form four small round membranes, as observed in wild-type cells (Fig. 1C; see Movie S2 in the supplemental material). However, prospore membrane extension stopped at this stage and extension to tube-like structures was never observed. This phenotype was similar to that of *spo71∆* mutant cells (Fig. 1C; see Movie S3 in the supplemental material). These results indicated that a dysferlin domain-only protein, Spo73, is involved in prospore membrane extension.

10.1128/mSphere.00038-15.2 Movie S1 Time-lapse video microscopy of prospore membranes, part 1. AN120 (wild type) cells expressing *GFP-SPO20^51-91^* were sporulated and analyzed by time-lapse fluorescence microscopy. The movie is shown at 5 frames/s, and frames were taken at 2-min intervals. A *z*-stack of 12 images was collected at each time point. Each frame is a projection of the images. Time is indicated in minutes. Download Movie S1, AVI file, 1.4 MB. Copyright © 2015 Okumura et al. 2015 Okumura et al. This content is distributed under the terms of the Creative Commons Attribution 4.0 International license.

10.1128/mSphere.00038-15.3 Movie S2 Time-lapse video microscopy of prospore membranes, part 2. TC545 (*spo73*∆ mutant) cells expressing *GFP-SPO20^51-91^* were sporulated and analyzed by time-lapse fluorescence microscopy. The movie is shown at 5 frames/s, and frames were taken at 2-min intervals. A *z*-stack of 12 images was collected at each time point. Each frame is a projection of the images. Time is indicated in minutes. Download Movie S2, AVI file, 1.7 MB. Copyright © 2015 Okumura et al. 2015 Okumura et al. This content is distributed under the terms of the Creative Commons Attribution 4.0 International license.

10.1128/mSphere.00038-15.4 Movie S3 Time-lapse video microscopy of prospore membranes, part 3. TC581 (*spo71*∆ mutant) cells expressing *GFP-SPO20^51-91^* were sporulated and analyzed by time-lapse fluorescence microscopy. The movie is shown at 5 frames/s, and frames were taken at 2-min intervals. A *z*-stack of 12 images was collected at each time point. Each frame is a projection of the images. Time is indicated in minutes. Download Movie S3, AVI file, 2 MB. Copyright © 2015 Okumura et al. 2015 Okumura et al. This content is distributed under the terms of the Creative Commons Attribution 4.0 International license.

### Capture of the nuclei by prospore membranes is partially defective in *spo73∆* mutant cells.

**FIG 2:**
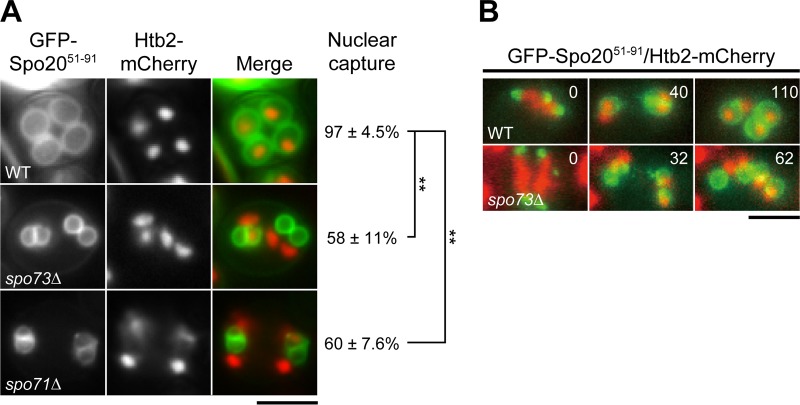
Analysis of nucleus capture in wild-type (WT) and *spo73∆* and *spo71∆* mutant cells. (A) AN120 (wild-type), TC545 (*spo73∆* mutant), and TC581 (*spo71∆* mutant) cells expressing *GFP-SPO20^51-91^* and *HTB2-mCherry* were sporulated for 8 to 9 h, and nuclear capture by prospore membranes was observed. Representative pictures are shown. More than 50 prospore membranes were examined in three independent colonies of each strain, and the percentage of prospore membranes that successfully captured each nucleus is shown as the mean value ± the standard deviation. **, *P* < 0.01 (Dunnett’s test). (B) AN120 (wild-type) and TC545 (*spo73∆* mutant) cells expressing *GFP-SPO20^51-91^* and *HTB2-mCherry* were sporulated for 6.5 h and subjected to time-lapse microscopic analysis. The time after the start of observation is shown in minutes. Scale bars, 5 µm.

Small prospore membranes in *spo73∆* mutant cells sometimes failed to capture each nucleus, although it has been reported that small prospore membranes in *spo71∆* mutant cells capture each nucleus efficiently (11). Capture of nuclei by prospore membranes was examined in wild-type and *spo73∆* mutant cells. Histone 2B tagged with mCherry was used as a nuclear marker and observed with a prospore membrane marker during sporulation. In contrast to wild-type cells, in which the vast majority of the nuclei were captured by prospore membranes, 42% of the prospore membranes failed to capture nuclei in *spo73∆* mutant cells (Fig. 2A). In addition, we found that, in contrast to a previous report, 40% of the prospore membranes failed to capture nuclei in *spo71∆* mutant cells (Fig. 2A). To confirm that the *spo73∆* mutant has a defect in nuclear capture, time-lapse analysis was performed. While nuclear capture occurred efficiently in wild-type cells (Fig. 2B; see Movie S4 in the supplemental material), in *spo73∆* mutant cells, we easily found cells in which prospore membranes failed to capture nuclei (Fig. 2B; see Movie S5). In these cells, the nuclei, marked by Htb2-mCherry, stuck at the leading edge of the prospore membrane and could not be engulfed. Thus, *spo73∆* mutant cells have a partial defect in nuclear capture by prospore membranes, and a similar phenotype is also seen in *spo71∆* mutant cells*.*

10.1128/mSphere.00038-15.5 Movie S4 Time-lapse series of nuclei and prospore membranes, part 1. AN120 (wild type) cells expressing *GFP-SPO20^51-91^* and *HTB2-mCherry* were sporulated and analyzed by time-lapse fluorescence microscopy. The movies are shown at 5 frames/s, and frames were taken at 2-min intervals. A *z*-stack of images was collected at each time point. Each frame is a projection of images. Time is indicated in minutes. Download Movie S4, AVI file, 4.5 MB. Copyright © 2015 Okumura et al. 2015 Okumura et al. This content is distributed under the terms of the Creative Commons Attribution 4.0 International license.

10.1128/mSphere.00038-15.6 Movie S5 Time-lapse series of nuclei and prospore membranes, part 2. TC545 (*spo73∆* mutant) cells expressing *GFP-SPO20^51-91^* and *HTB2-mCherry* were sporulated and analyzed by time-lapse fluorescence microscopy. The movies are shown at 5 frames/s, and frames were taken at 2-min intervals. A *z*-stack of images was collected at each time point. Each frame is a projection of images. Time is indicated in minutes. Download Movie S5, AVI file, 4.7 MB. Copyright © 2015 Okumura et al. 2015 Okumura et al. This content is distributed under the terms of the Creative Commons Attribution 4.0 International license.

### Phenotype of the *spo73∆* mutant is similar to that of *spo71∆* and *vps13∆* mutants.

**FIG 3:**
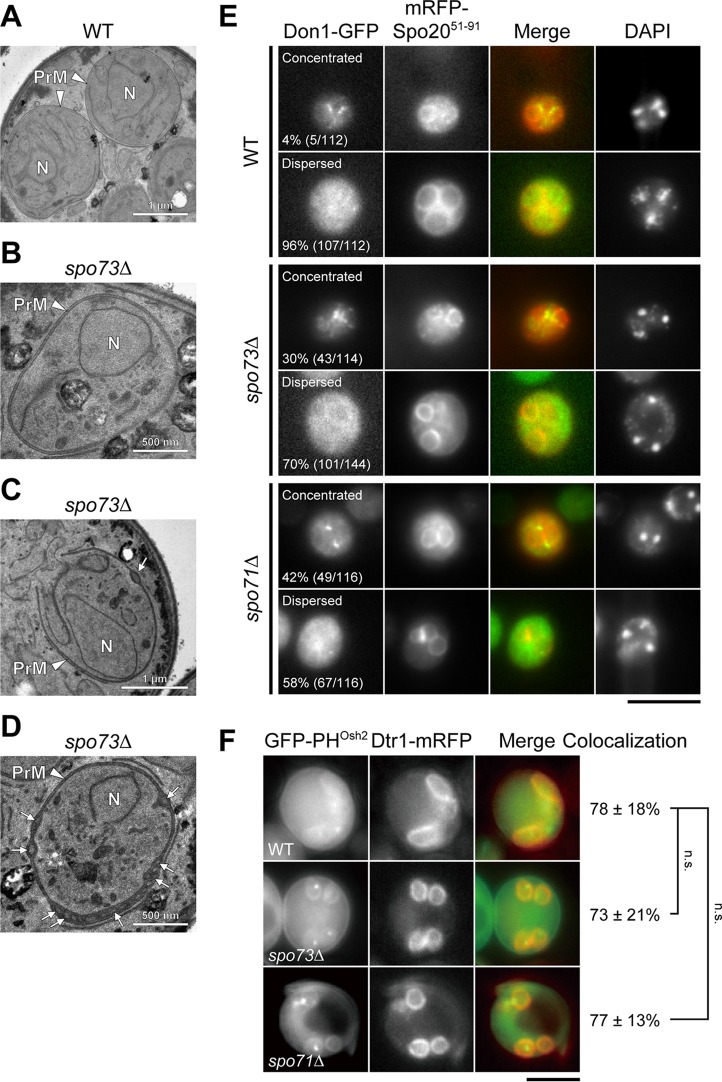
Phenotypic analysis of *spo73∆* mutant. (A to D) AN120 (wild-type [WT], A) and TC545 (*spo73∆* mutant, B to D) cells were sporulated for 7.5 h and subjected to EM analysis. Prospore membranes and intraluminal vesicles are indicated by arrowheads and arrows, respectively. N, nucleus; PrM, prospore membrane. A scale bar is shown in each panel. (E) YUY088 (*DON1-GFP*), YUY090(*DON1*-*GFP spo73*∆), and YUY089 (*DON1-GFP spo71*∆) cells expressing *mRFP-SPO20^51-91^* were sporulated for 9 to 11 h to analyze postmeiotic cells selected with DAPI staining. Representative cells are shown. Upper panels for each strain show cells in which Don1 was concentrated on the prospore membrane. Lower panels for each strain show cells in which Don1 was dispersed in the cytoplasm localization after removal from the leading edge. Scale bar, 5 µm. The percentage of cells showing each pattern is shown. More than 100 prospore membranes were examined for each strain. (F) AN120 (wild-type), TC545 (*spo73*∆ mutant), and TC581 (*spo71*∆ mutant) cells expressing *DTR1-mRFP* and *GFP-PH^OSH2^* were sporulated for 7.5 to 9 h and observed. Representative cells are shown. Scale bar, 5 µm. The average percentage of GFP-PH*^OSH2^*-positive prospore membranes in Dtr1-mRFP-positive prospore membranes is shown with the standard deviation. More than 50 prospore membranes were scored in three separate experiments. n.s., not significant (Dunnett’s test).

We saw similar defects in prospore membrane extension and nuclear capture in *spo73∆* and *spo71∆* mutant cells. *spo71∆* mutant cells have been shown to form prospore membranes with bubbles or intraluminal vesicles between double membranes, have a partial defect in removal of the LEC, and form prospore membranes with decreased levels of PI4P (12). Thus, we examined *spo73∆* mutant cells for these phenotypes. First, we performed electron microscopy (EM) analysis. Wild-type and *spo73∆* mutant cells were sporulated and analyzed by EM. In wild-type cells, all of the prospore membranes looked to be closely apposed double membranes except for those in cells with apparent spore walls (Fig. 3A). In contrast, about 40% (68 in 167) of *spo73∆* mutant prospore membranes showed bubbles or intraluminal vesicles (Fig. 3B to D), which have previously been reported in *spo71∆* and *vps13∆* mutant cells (12). Next, we assessed the removal of the LEC, which is required for prospore membrane closure. Postmeiotic cells selected by 4′,6-diamidino-2-phenylindole (DAPI) staining were observed to detect LECs by using Don1-GFP as a marker. While most LECs were removed in wild-type postmeiotic cells, the LECs of 30% of the prospore membranes were still present in postmeiotic *spo73∆* mutant cells, suggesting a partial defect in prospore membrane closure (Fig. 3E). Finally, PI4P levels at the prospore membrane were examined by using GFP-PH^OSH2^ (29) as a PI4P biosensor and Dtr1 fused to red fluorescent protein (RFP) (9) as a prospore membrane marker. Although the original procedure was mostly reproducible, we revised the experimental procedure to observe sporulating cells at 7 to 9 h at 30°C to focus on cells around the time of prospore membrane formation. In all three strains, about 80% of the RFP-positive prospore membranes were GFP positive, indicating that PI4P levels were not changed in wild-type and *spo73∆* and *spo71∆* mutant cells (Fig. 3F). This is in contrast to an earlier report indicating that PI4P levels are reduced in *spo71∆* mutant cells (12). The reason for this discrepancy is unclear; nonetheless, these results indicate that the *spo73∆* mutant has prospore membrane extension defects similar to those of the *spo71∆* and *vps13∆* mutants.

### Spo73 localizes to the prospore membrane independently of Spo71 and Vps13.

**FIG 4:**
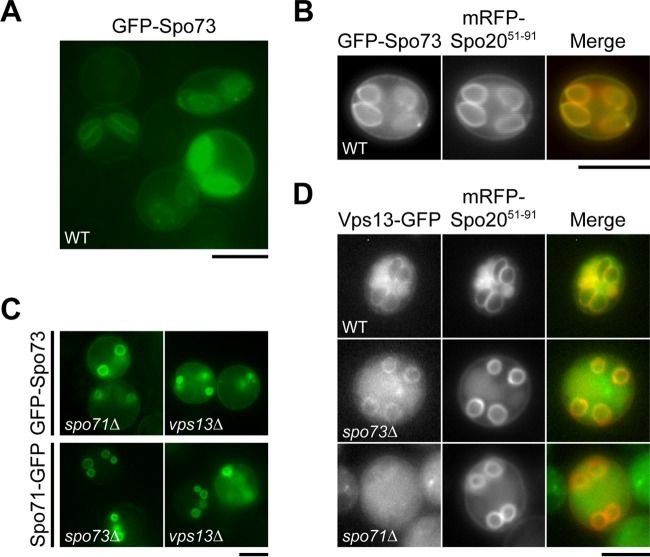
Localization of Spo73 during sporulation and its relationship to Vps13 and Spo71. (A) AN120 (wild-type [WT]) cells were transformed with 424-P_SPO20_-GFP-SPO73, sporulated, and observed at 7.5 h. (B) AN120 (wild-type) cells expressing *GFP-SPO73* and *mRFP-SPO20^51-91^* were sporulated for 7.5 h and observed. A representative cell is shown. (C) TC581 (*spo71∆* mutant) and HI29 (*vps13∆* mutant) cells were transformed with 424-P_SPO20_-GFP-SPO73. TC545 (*spo73∆* mutant) and HI29 (*vps13∆* mutant) cells were transformed with 424-SPO71-GFP. Strains were sporulated for 7.5 h and observed. (D) ICY10 (*VPS13-GFP*), TC578 (*VPS13-GFP spo73∆*), and TC577 (*VPS13-GFP spo71∆*) cells expressing *mRFP-SPO20^51-91^* were sporulated for 7.5 h and observed. Scale bars, 5 µm.

Coluccio et al. have shown that hemagglutinin-tagged Spo73 localizes to dots on prospore membranes in fixed cells (19). Subcellular localization of Spo73 protein was observed in live cells by using N-terminally GFP-tagged Spo73 expressed from a low-copy-number vector. Although the plasmid rescued the sporulation defect of the *spo73∆* mutant, no apparent localization pattern was observed (data not shown). Therefore, GFP-Spo73 was overexpressed during sporulation by using a multicopy vector. Upon overexpression, a clear prospore membrane pattern was observed (Fig. 4A). Colocalization with a prospore membrane marker confirmed that Spo73 localizes to prospore membranes (Fig. 4B).

Vps13 localization to prospore membranes is dependent on Spo71 (12). Thus, interdependency of localization among Spo73, Spo71, and Vps13 was analyzed. A GFP-tagged version of each gene was expressed in mutants with the other two genes deleted and examined during sporulation. Spo73-GFP localized to prospore membranes in *spo71∆* and *vps13∆* mutant cells (Fig. 4C), indicating that Spo73 localization to the prospore membrane is not dependent on Spo71 or Vps13. Similarly, prospore membrane localization of Spo71-GFP was not disturbed in *spo73∆* and *vps13∆* mutant cells (Fig. 4C), indicating that Spo71 localization to the prospore membrane is not dependent on Spo73 and Vps13. As to Vps13-GFP, although some cytosolic background fluorescence was observed, a prospore membrane pattern was observed in *spo73∆* mutant cells (Fig. 4D). This indicates that localization of Vps13 to the prospore membrane is not dependent on Spo73, which is distinct from the dependence of Vps13 localization on Spo71.

### Mutations in a surface patch of basic amino acid residues of Spo73 disrupt its localization to the prospore membrane.

**FIG 5:**
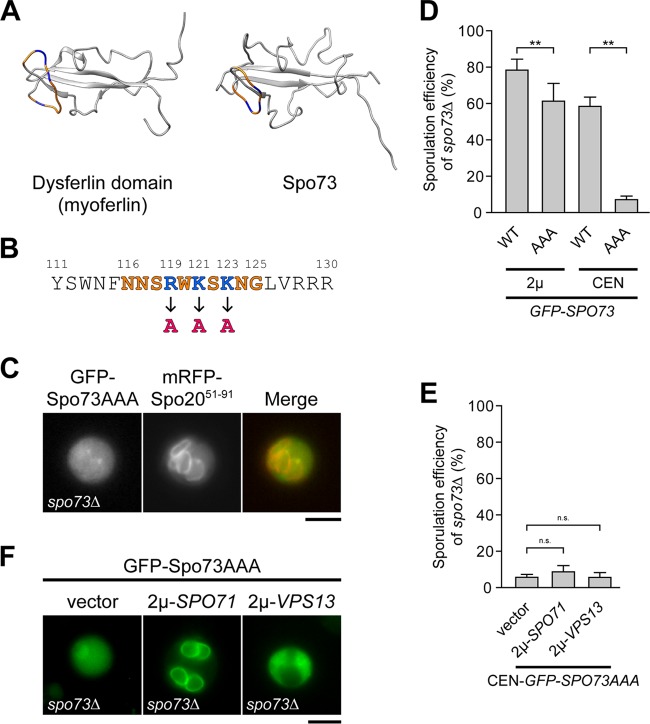
3D modeling of Spo73 and analysis of Spo73AAA. (A) 3D model of Spo73 and internal dysferlin domain of myoferlin (PDB code 2K2O A chain) are shown. Conserved loop structures are orange. Basic amino acids in the loop are blue. (B) Amino acid sequence of Spo73 around the basic loop showing the mutations introduced to produce Spo73AAA. Amino acid numbers are shown. (C) TC545 (*spo73∆* mutant) cells carrying 424-P_SPO20_-GFP-SPO73AAA and 426-*mRFP-SPO20^51-91^* were sporulated for 7 h and observed. Scale bar, 5 µm. (D) TC545 (*spo73∆* mutant) cells carrying 424-P_SPO20_-GFP-SPO73 (2μ-GFP-SPO73WT), 424-P_SPO20_-GFP-SPO73AAA (2μ-GFP-SPO73AAA), 314-P_SPO73_-GFP-SPO73 (CEN-GFP-SPO73WT), or 314-P_SPO73_-GFP-SPO73AAA (CEN-GFP-SPO73AAA) were sporulated. Sporulation efficiency is shown as the mean ± the standard deviation. More than 200 cells were examined in 10 independent colonies of each strain. **, *P* < 0.01 (Tukey-Kramer test). (E) TC545 (*spo73∆* mutant) carrying 314-P_SPO73_-GFP-SPO73AAA (CEN-GFP-SPO73AAA) was transformed with pRS426 (vector), 426-SPO71 (2μ-SPO71), or 426-VPS13 (2μ-VPS13). The sporulation efficiency of resulting strains is shown as the mean ± the standard deviation. More than 200 cells were examined in six independent colonies of each strain, except five of the strain carrying 426-VPS13. n.s., not significant (Dunnett’s test). (F) TC545 (*spo73∆* mutant) cells carrying 424-P_SPO20_-GFP-SPO73AAA (2μ-GFP-SPO73AAA) and 426-SPO71 (2μ-SPO71), 426-VPS13 (2μ-VPS13), or pRS426 (vector) were sporulated for 7 h and observed. Representative cells are shown. Scale bar, 5 µm.

Our analysis revealed that Spo73 consists mostly of the dysferlin domain. The three-dimensional (3D) structure of Spo73 was modeled on the structure of the dysferlin domain of myoferlin (Protein Data Bank [PDB] code 2K2O A chain) (30) (Fig. 5A). A similar 3D model was obtained on the basis of the 3D structure of the dysferlin domain of dysferlin (PDB code 4CAH B chain; data not shown) (23). Besides the antiparallel β-sheet structure formed by the N- and C-terminal dysferlin domains, a surface basic loop was found in both dysferlin domains, which may be important for electrostatic interactions with acidic materials, such as protein surfaces or phospholipids. Thus, we mutated those amino acids in GFP-Spo73 to alanines (R119A, K121A, K123A) (Fig. 5B). The mutant version of GFP-Spo73 (GFP-Spo73AAA) was overexpressed in *spo73∆* mutant cells and observed during sporulation. GFP-Spo73AAA-expressing cells did not show the prospore membrane pattern; instead, they showed a uniform cytosolic pattern (Fig. 5C). However, this strain sporulated (61%) at a level only slightly lower than that of the *spo73∆* mutant strain overexpressing GFP-Spo73 (78%), suggesting that the overall structure of Spo73 is not disrupted by the mutations and that overexpression may be masking a more severe sporulation defect. Therefore, we expressed GFP-Spo73AAA from a centromere (CEN)-based low-copy-number vector by using its own promoter. This strain sporulated to a significantly lesser extent (7%) than the strain expressing wild-type GFP-Spo73 from a low-copy-number vector (58%) (Fig. 5D). These results indicate that the basic loop of Spo73 is required for its localization to the prospore membrane and that *spo73AAA* is partially functional.

### Overexpression of Spo71 can localize Spo73AAA to the prospore membrane.

Taking advantage of *spo73AAA*, which is partially functional, we examined the relationship between Spo73 and Spo71 or Vps13. Suppression of *spo73AAA* was tested by using cells expressing *spo73AAA* from a CEN-based low-copy-number vector. The *SPO71* and *VPS13* genes were each overexpressed by using a 2µm-based vector, and percent sporulation was examined. Also, the effect on the localization of Spo73AAA was tested by using cells overexpressing GFP-Spo73AAA and Spo71 or Vps13 from 2µm-based vectors. We could not detect apparent suppression of *spo73AAA* by overexpression of *SPO71* or *VPS13* (Fig. 5E). However, in contrast to the cytosolic pattern observed in cells overexpressing only GFP-Spo73AAA or both GFP-Spo73AAA and Vps13, an apparent prospore membrane pattern was observed in cells overexpressing both GFP-Spo73AAA and Spo71 (Fig. 5F). Considering a previous report showing that Spo71-GFP localizes to the prospore membrane, which was detectable only when overexpressed (12), the present observation suggests that overexpression of Spo71 can recruit detectable levels of mutant GFP-Spo73 to the prospore membrane. The results also imply that the function of Spo73 in prospore membrane extension is not necessarily dependent on its localization to the prospore membrane.

### *SPO73* has a genetic interaction with *SPO1* and *SMA2.*

**FIG 6:**
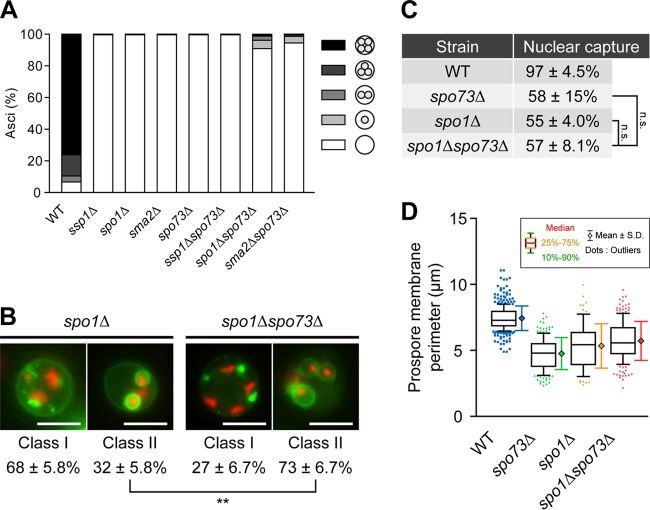
Analysis of a genetic interaction of *SPO73* with the LEC and the spore membrane-bending pathway genes. (A) AN120 (wild-type [WT]), NY551H (*ssp1*∆ mutant), TC554 (*spo1*∆ mutant), HI44 (*sma2*∆ mutant), TC555 (*spo73*∆ mutant), TC562H (*ssp1*∆ *spo73*∆ mutant), TC568 (*spo1*∆ *spo73*∆ mutant), and TC569 (*sma2*∆ *spo73*∆ mutant) cells were sporulated for 24 h and observed by DIC microscopy. Sporulation percentages are shown in the diagram. More than 200 cells of each strain were observed. (B) TC554 (*spo1∆* mutant), and TC568 (*spo1∆ spo73∆* mutant) cells expressing *GFP-SPO20^51-91^* and *HTB2-mCherry* were sporulated and analyzed for the absence (class I) or presence (class II) of prospore membranes. Representative images of postmeiotic cells of each mutant are shown. Classification of the phenotype is described in the text. More than 50 GFP- and mCherry-positive cells in three independent colonies were classified, and the percentage of each class is shown as the mean ± the standard deviation. **, *P* < 0.01 (Student’s *t* test). (C) Percentages of nuclear capture by prospore membranes formed were counted in AN120 (wild-type), TC555 (*spo73∆* mutant), TC554 (*spo1∆* mutant), and TC568 (*spo1∆ spo73∆* mutant) cells as described in the legend to Fig. 2A. n.s., not significant (Tukey-Kramer test). (D) Prospore membrane perimeters of postmeiotic cells of strains AN120 (wild-type), TC555 (*spo73∆* mutant), TC554 (*spo1∆* mutant), and TC568 (*spo1∆ spo73∆* mutant) are shown in a box-and-whisker plot. The horizontal line within the box indicates the median, the boundaries of the box indicate the 25th and 75th percentiles, and the whiskers indicate the 10th and 90th percentiles. Points below and above the whiskers are drawn as individual dots. More than 150 prospore membranes of each strain were measured. The bar to the right of each box shows the mean ± the standard deviation.

Parodi et al. showed that *SPO71* has a weak genetic interaction with *SPO1* (11). Also, although no data were shown, a genetic interaction between *SPO73* and *SPO1* has been described (31). Therefore, we looked for a genetic interaction of *SPO73* with *SPO1* and *SMA2*, which code for proteins involved in the spore membrane-bending pathway, together with *SSP1*, which codes for the essential component of the LEC. While all of the single mutants, i.e., the *spo1∆*, *sma2∆*, and *spo73∆* mutants, were completely sporulation defective, the *spo1∆ spo73∆* and *sma2∆ spo73∆* double mutants sporulated reproducibly (*spo1∆ spo73∆* mutant, 9%; *sma2∆ spo73∆* mutant, 5%) (Fig. 6A). No sporulation of the *ssp1∆ spo73∆* double mutant was observed.

To assess the mechanism of suppression, the phenotype of the *spo1∆ spo73∆* double mutant was analyzed in comparison with that of single mutants. As reported by Parodi et al. (11), Htb2-mCherry and GFP-Spo20^51-91^ were expressed in the mutant cells during sporulation, and postmeiotic cells were classified into two classes. Cells without apparent prospore membranes were designated class I. Cells with at least one prospore membrane were designated class II. In addition, the capture of the nuclei by prospore membranes and prospore membrane perimeters was assessed. As is the case with the *spo71∆* mutant, the *spo73∆* mutant partially suppressed the defect of the *spo1∆* mutant. The *spo1∆ spo73∆* double mutant exhibited more cells in class II, indicating a defect less severe than that of the *spo1∆* mutant (Fig. 6B). No apparent suppression of the nuclear capture rate was observed (Fig. 6C). As to prospore membrane size, the *spo73∆* mutant cells showed a small prospore membrane phenotype; however, apparent suppression was not observed in the *spo1∆ spo73∆* double mutant (Fig. 6D). But we found some prospore membranes in the double mutant larger than those in the *spo73∆* mutant, consistent with the 9% sporulation of this mutant. Our results indicate that *SPO73* genetically interacts with the genes involved in spore membrane bending, similar to *SPO71*.

## DISCUSSION

Previous studies have shown the involvement of Vps13 and Spo71 in prospore membrane extension (9, 11, 12); however, the overall mechanism of the process has remained to be elucidated. Here, we identified Spo73 as an additional factor involved in this process.

The *spo73∆* mutant showed phenotypes similar to those of the *spo71∆* and *vps13∆* mutants; it formed small prospore membranes with intraluminal vesicles, was partially defective in nuclear capture by prospore membranes, and was also partially defective in the removal of the LEC. All three proteins localized to the prospore membrane. Analyses of interaction and localization dependency in a previous study showed that Spo71 interacts with and recruits Vps13 to the prospore membrane (12). Our analysis showed that localization of Spo73 to the prospore membrane is not dependent on Spo71 and Vps13 and vice versa. However, a mutant Spo73 protein that showed a cytosolic localization pattern was targeted to the prospore membrane when Spo71 was overexpressed. Although no genetic suppression was detected, this rescue of localization could be explained by interaction between Spo73 and Spo71. Further analysis is required to show a physical interaction between these proteins.

Membrane-bending pathway mutants form wide and aberrant prospore membranes (14), which are quite the opposite of the small prospore membranes formed by the *spo73∆* mutant. A genetic interaction was observed between *SPO73* and the genes involved in spore membrane bending, *SPO1* and *SMA2*. Our analysis of prospore membranes showed that the *spo1∆* mutant phenotype was partially suppressed by *spo73∆*, although apparent suppression of the *spo73∆* mutant phenotype by *spo1∆* was not observed. *SPO71* has a genetic interaction with *SPO1* as well (11), although it is weaker than the interaction between *SPO73* and *SPO1* or *SMA2*. Thus, our results support the idea that Spo73 functions with Spo71 in prospore membrane extension and that this function somehow works to counteract the bending force exerted by the spore membrane-bending pathway.

In our results, the rate of prospore membrane capture in the mutants was somewhat lower than reported (11). This could be caused by a difference in the strain background or some experimental conditions.

It has been reported that PI4P levels on prospore membranes of *vps13∆* and *spo71∆* mutant cells are lower than on those of wild-type cells (9, 12). Thus, Vps13 and Spo71 are considered to have a role in regulating the PI4P levels on prospore membranes. This was based on the observation of a PI4P biosensor in cells that sporulated overnight at room temperature, which could contain cells at various stages during sporulation. To focus on cells just around meiosis, we changed the method to sporulating the cells at 30°C for 7 to 9 h. Using this new method, we could not detect any apparent difference in PI4P levels in *spo71∆* and *spo73∆* mutant cells from those in wild-type cells, suggesting that PI4P levels are not affected or are affected at undetectable levels in those mutants around meiosis II. The reasons for the discrepancy between the observations in this and previous studies are unclear, but one of the reasons could be that observing cells at later stages amplified the difference to detectable levels in the original procedure.

Bioinformatic analysis revealed that Spo73 is a dysferlin domain-only protein. Dysferlin domains are found in a subgroup of the ferlin family proteins (20). In humans, three out of the six ferlin family proteins contain dysferlin domains, including dysferlin and myoferlin. Dysferlin is a protein considered to function in Ca^2+^-dependent membrane repair in muscles, and mutations in its gene cause dysferlinopathies, including limb girdle muscular dystrophy type 2B and Miyoshi myopathy (21–23). Myoferlin is involved in myoblast fusion (32). In *S. cerevisiae*, dysferlin domains are found in a subset of peroxins, Pex30, Pex31, and Pex32, that function in the control of peroxisome size and number (33). All of the dysferlin domain-containing proteins analyzed so far are involved in membrane-related processes, although the precise role of this domain is still unknown. In this study, we revealed that dysferlin domain-only protein Spo73 is required for proper prospore membrane formation. Prospore membrane formation of *S. cerevisiae* is a process of dynamic reorganization of intracellular membrane structure and has been studied as a model of *de novo* membrane formation. Given that other dysferlin domain proteins contain specific domains in addition to this domain (28), dysferlin domain-only protein Spo73 is the most suitable protein to elucidate the conserved function of dysferlin domains.

Our analysis revealed that a mutant Spo73 protein that has mutations in a conserved basic loop cannot localize to the prospore membrane. Thus, Spo73 may interact with acidic materials such as phospholipids on the prospore membrane through its conserved basic loop. Although more analysis is required, interaction with membranes could be an important feature of the dysferlin domain. Interestingly, one of the mutations causing muscular dystrophy resides in the basic loop of the inner dysferlin domain of the dysferlin protein (R1022Q) (23), suggesting that this mutation could affect the interaction of the domain with the membrane, which could cause the disease phenotype. Further analysis of Spo73 could provide insights into the conserved function of dysferlin domains and the pathogenesis of dysferlinopathy.

## MATERIALS AND METHODS

### Yeast strains and media.

Standard media and genetic techniques were used unless otherwise noted (34). The genes corresponding to the mutants selected for screening from the deletion collection in the BY4743 background (Invitrogen, San Diego, CA) are listed in Table S1 in the supplemental material. Other yeast strains used in this study are listed in Table 1. All of the strains in Table 1 were derived from the SK1 background. PCR-based gene alterations were performed as previously described (35). Strains were constructed with the primers and plasmids in Table S2. The primers used in this study are listed in Table S3. All PCR-based integrations and disruptions were confirmed by genomic PCR. All of the deletions constructed in this study were confirmed to be rescuable by expression of the genes deleted.

**TABLE 1:** Yeast strains used in this study

Strain	Genotype	Source or reference
AN117-4B	*MAT*α *ura3 leu2 his3*Δ*SK trp1*::*hisG arg4-NspI lys2 ho*Δ::*LYS2 rme1*Δ::*LEU2*	35
AN117-16D	*MAT***a** *ura3 leu2 his3*Δ*SK trp1*::*hisG lys2 ho*Δ::*LYS2*	35
AN120	*MAT***a**/*MAT*α *ura3*/*ura3 his3*Δ*SK*/*his3*Δ*SK trp1*::*hisG*/*trp1*::*hisG ARG4*/*arg4-NspI lys2*/*lys2 ho*Δ::*LYS2*/*ho*Δ::*LYS2 RME1*/*rme1*Δ::*LEU2 leu2*/*leu2*	35
TC581	*MAT***a**/*MAT*α *ura3*/*ura3 his3*Δ*SK*/*his3*Δ*SK trp1*::*hisG*/*trp1*::*hisG ARG4*/*arg4-NspI lys2*/*lys2 ho*Δ::*LYS2*/*ho*Δ::*LYS2 RME1*/*rme1*Δ::*LEU2 leu2*/*leu2 spo71*Δ::*kanMX6*/*spo71*Δ::*kanMX6*	This study
TC545	*MAT***a**/*MAT*α *ura3*/*ura3 his3*Δ*SK*/*his3*Δ*SK trp1*::*hisG*/*trp1*::*hisG ARG4*/*arg4-NspI lys2*/*lys2 ho*Δ::*LYS2*/*ho*Δ::*LYS2 RME1*/*rme1*Δ::*LEU2 leu2*/*leu2 spo73*Δ::*kanMX6*/*spo73*Δ::*kanMX6*	This study
YUY088	*MAT***a**/*MAT*α *his3*Δ*SK*/*his3*Δ*SK trp1*::*hisG*/*trp1*::*hisG ARG4*/*arg4-NspI lys2*/*lys2 ho*Δ::*LYS2*/*ho*Δ::*LYS2 RME1*/*rme1*Δ::*LEU2 leu2*/*leu2 ura3*/*ura3*::*URA3-DON1-GFP*	This study
YUY089	*MAT***a**/*MAT*α *his3*Δ*SK*/*his3*Δ*SK trp1*::*hisG*/*trp1*::*hisG ARG4*/*arg4-NspI lys2*/*lys2 ho*Δ::*LYS2*/*ho*Δ::*LYS2 RME1*/*rme1*Δ::*LEU2 leu2*/*leu2 ura3*/*ura3*::*URA3-DON1-GFP spo71*Δ::*kanMX6*/*spo71*Δ::*kanMX6*	This study
YUY090	*MAT***a**/*MAT*α *his3*Δ*SK*/*his3*Δ*SK trp1*::*hisG*/*trp1*::*hisG ARG4*/*arg4-NspI lys2*/*lys2 ho*Δ::*LYS2*/*ho*Δ::*LYS2 RME1*/*rme1*Δ::*LEU2 leu2*/*leu2 ura3*/*ura3*::*URA3-DON1-GFP spo73*Δ::*kanMX6*/*spo73*Δ::*kanMX6*	This study
HI29	*MAT***a**/*MAT*α *ura3*/*ura3 his3*Δ*SK*/*his3*Δ*SK trp1*::*hisG*/*trp1*::*hisG ARG4*/*arg4-NspI lys2*/*lys2 ho*Δ::*LYS2*/*ho*Δ::*LYS2 RME1*/*rme1*Δ::*LEU2 leu2*/*leu2 vps13*Δ::*kanMX6*/*vps13*Δ::*kanMX6*	8
TC554	*MAT***a**/*MAT*α *ura3*/*ura3 his3*Δ*SK*/*his3*Δ*SK trp1*::*hisG*/*trp1*::*hisG ARG4*/*arg4-NspI lys2*/*lys2 ho*Δ::*LYS2*/*ho*Δ::*LYS2 RME1*/*rme1*Δ::*LEU2 leu2*/*leu2 spo1*Δ::*HIS3MX6*/*spo1*Δ::*HIS3MX6*	This study
TC555	*MAT***a**/*MAT*α *ura3*/*ura3 his3*Δ*SK*/*his3*Δ*SK trp1*::*hisG*/*trp1*::*hisG ARG4*/*arg4-NspI lys2*/*lys2 ho*Δ::*LYS2*/*ho*Δ::*LYS2 RME1*/*rme1*Δ::*LEU2 leu2*/*leu2 spo73*Δ::*HIS3MX6*/*spo73*Δ::*HIS3MX6*	This study
HI42	*MAT*α *ura3 leu2 his3*Δ*SK trp1*::*hisG arg4-NspI lys2 ho*Δ::*LYS2 rme1*Δ::*LEU2 sma2*Δ::*HIS3MX6*	8
HI43	*MAT***a** *ura3 leu2 his3*Δ*SK trp1*::*hisG lys2 ho*Δ::*LYS2 sma2*Δ::*HIS3MX6*	8
HI44	*MAT***a**/*MAT*α *ura3*/*ura3 his3*Δ*SK*/*his3*Δ*SK trp1*::*hisG*/*trp1*::*hisG ARG4*/*arg4-NspI lys2*/*lys2 ho*∆::*LYS2*/*ho*∆::*LYS2 RME1*/*rme1*∆::*LEU2 leu2*/*leu2 sma2*∆::*HIS3MX6/sma2*∆::*HIS3MX6*	8
TC568	*MAT***a**/*MAT*α *ura3*/*ura3 his3* ∆*SK*/*his3∆SK trp1*::*hisG/trp1*::*hisG ARG4*/*arg4-NspI lys2/lys2 ho*∆::*LYS2/ho*∆::*LYS2 RME1*/*rme1*∆::*LEU2 leu2/leu2 spo1*∆::*HIS3MX*6/*spo1*∆::*HIS3MX6/spo73*∆::*kanMX6*/*spo73*∆::*kanMX6*	This study
TC569	*MAT***a**/*MAT*α *ura3/ura3 his3 ∆SK/his3∆SK trp1*::*hisG/trp1*::*hisG ARG4/arg4*-*NspI lys2/lys2 ho*∆::*LYS2*/*ho*∆::*LYS2 RME1/rme1*∆::*LEU2 leu2/leu2 sma2*∆::*HIS3MX6*/*sma2*∆::*HIS3MX6 spo73*∆::*kanMX6*/*spo73*∆::*kanMX6*	This study
NY551H	*MAT***a**/*MAT*α *ura3/ura3 HIS3/his3∆SK trp1*::*hisG/trp1*::*hisG ARG4*/*arg4-NspI lys2/lys2 ho*∆::*LYS2/ho∆*::*LYS2 RME1/rme1∆*::*LEU2 leu2/leu2 ssp1*∆::*kanMX6/ssp1∆*::*kanMX6*	This study
TC562H	*MAT***a**/*MAT*α *ura3/ura3 HIS3/his3∆SK trp1*::*hisG/trp1*::*hisG ARG4/arg4-NspI lys2/lys2 ho*∆::*LYS2/ho∆*::*LYS2 RME1/rme1∆*::*LEU2 leu2/leu2 ssp1∆*::*kanMX6 /ssp1*∆::*kanMX6 spo73∆*::*kanMX6/spo73*∆::*kanMX6*	This study
ICY10	*MAT***a**/*MAT*α *ura3/ura3 his3 ∆SK/his3∆SK trp1*::*hisG/trp1*::*hisG ARG4/arg4-NspI lys2/lys2 ho*∆::*LYS2/ho*∆::*LYS2 RME1/rme1*∆::*LEU2 leu2/leu2 VPS13*::*GFP-HIS3MX6/VPS13*::*GFP-HIS3MX6*	This study
TC577	*MAT***a**/*MAT*α *ura3/ura3 his3 ∆SK*/*his3∆SK trp1*::*hisG/trp1*::*hisG ARG4/arg4-NspI lys2/lys2 ho*∆::*LYS2/ho*∆::*LYS2 RME1*/*rme1*∆::*LEU2 leu2/leu2 VPS13*::*GFP-HIS3MX6/VPS13*::*GFP-HIS3MX6 spo71*∆::*kanMX6/spo71*∆::*kanMX6*	This study
TC578	*MAT***a**/*MAT*α *ura3/ura3 his3∆SK*/*his3∆SK trp1*::*hisG/trp1*::*hisG ARG4/arg4*-*NspI lys2/lys2 ho*∆::*LYS2*/*ho∆*::*LYS2 RME1*/*rme1∆*::*LEU2 leu2/leu2 VPS13*::*GFP-HIS3MX6*/*VPS13*::*GFP-HIS3MX6 spo73∆*::*kanMX6/spo73∆*::*kanMX6*	This study

10.1128/mSphere.00038-15.7 Table S2 Construction of the strains used in this study. Download Table S2, PDF file, 0.06 MB. Copyright © 2015 Okumura et al. 2015 Okumura et al. This content is distributed under the terms of the Creative Commons Attribution 4.0 International license.

10.1128/mSphere.00038-15.8 Table S3 Primers used in this study. Download Table S3, PDF file, 0.03 MB. Copyright © 2015 Okumura et al. 2015 Okumura et al. This content is distributed under the terms of the Creative Commons Attribution 4.0 International license.

### Plasmids.

The plasmids used in this study are listed in Table S4 in the supplemental material. To generate 306-DON1-GFP, *DON1-GFP* was cut out of pSB8 (35) and cloned into pRS306. To construct pSB1 (pFA6a-His3MX6-P_SPO20_-GFP), the *GAL1* promoter of pFA6a-His3MX6-PGAL1-GFP (36) was replaced with the *SPO20* promoter (35). To generate 424-GFP-SPO73, the promoter region of a chromosomal copy of *SPO73* was replaced with a P_SPO20_-GFP fusion by a PCR-based method with primers OK13 and OK14 by using pSB1 as a template. *P_SPO20_-GFP-SPO73* was then amplified from genomic DNA by using HT369 and OK15 and cloned into pRS424. To construct 424-P_SPO20_-GFP-SPO73AAA, site-directed mutagenesis was performed by the QuikChange method with primers OK29 and OK30. To construct 314-P_SPO73_-GFP-SPO73 and 314-P_SPO73_-GFP-SPO73AAA, *GFP-SPO73* and *GFP-SPO73AAA* without a promoter were cloned into pRS314 together with a *SPO73* promoter fragment amplified with HT421 and HT422. To construct 426-SPO71 and 426-VPS13, *SPO71* and *VPS13* were amplified from SK1 genomic DNA by PCR with primers OK11 and HT424 and primers TN194 and TN197, respectively, and cloned into pRS426. To generate 316-HTB2-mCherry, a chromosomal copy of *HTB2* was fused to mCherry by PCR-mediated transformation. *HTB2-mCherry* was then amplified from genomic DNA with primers TN62 and HT66E and then cloned into pRS316. All amplified regions were sequenced.

10.1128/mSphere.00038-15.9 Table S4 Plasmids used in this study. Download Table S4, PDF file, 0.06 MB. Copyright © 2015 Okumura et al. 2015 Okumura et al. This content is distributed under the terms of the Creative Commons Attribution 4.0 International license.

### Sporulation.

Sporulation was performed as previously described (37). For sporulation on plates, cells grown on yeast extract-peptone-dextrose or synthetic dextrose medium plates were shifted to sporulation plates (1% potassium acetate) and incubated at 30°C.

### Light microscopy.

Differential interference contrast (DIC) images and fluorescence images were obtained with a BX71 microscope (Olympus, Tokyo, Japan), a Quantix 1400 camera (Photometrics, Tucson, AZ), and IPLab 3.7 software (Scanalytics, Fairfax, VA). Cells were fixed with 3.7% formaldehyde when required.

Nuclear capture counting and prospore membrane perimeter measurement were performed as previously described (11). Cells expressing both *HTB2-mCherry* and *GFP-SPO20^51-91^* were sporulated for 9 h, and postmeiotic cells were identified by the pattern of Htb2-mCherry localization. The percentage of prospore membranes capturing nuclei is shown for each strain. Prospore membrane perimeters were measured by ImageJ (http://rsb.info.nih.gov/ij/).

PI4P levels at the prospore membrane were tested as previously described (12), except that sporulation was induced for 7 to 9 h at 30°C. Cells carrying both pRS426-P_PRC1_ -GFP-PH*^OSH2^* and pRS424-*DTR1*-*RFP* were observed under the microscope, and GFP-PH*^OSH2^*/Dtr1-mRFP colocalization was quantified.

Time-lapse imaging was performed as previously described (38). Images were captured on a Zeiss Axiovert 100 microscope equipped with a CoolSNAP HQ camera (Photometrics) at 2-min intervals with IPLab 3.6.5a software (Scanalytics). The temperature was held at 28°C during image collection. 3D stacks were performed with IPLab 3.6.5a.

### EM.

Sporulating cells were collected, fixed with 2% paraformaldehyde–2% glutaraldehyde in 0.1 M phosphate buffer (pH 7.4) at 4°C overnight, and washed three times with 0.1 M phosphate buffer. Cells were then treated with 4% KMnO_4_ at room temperature for an hour, washed with distilled water, and embedded in 1.5% low-melting-point agarose. Samples were dehydrated with acetone and infiltrated with increasing concentrations of Spurr’s resin in propylene oxide and finally with 100% Spurr’s resin. After polymerization with dimethylaminoethanol, ultrathin sections were cut on a Leica Ultracut UCT microtome and stained with uranyl acetate and lead citrate. The sections were then viewed with a JEOL 2000EX electron microscope.

### 3D structure modeling.

HHpred (http://toolkit.tuebingen.mpg.de/hhpred) (39) was used to identify related structures of Spo73. The 3D model of Spo73 was constructed on the basis of the structure of the internal dysferlin domain of myoferlin by using Modeller (http://toolkit.tuebingen.mpg.de/modeller) and shown by using Chimera (http://www.cgl.ucsf.edu/chimera).
