# Supplementary material for: The Dysferlin Domain-Only Protein, Spo73, Is Required for Prospore Membrane Extension in Saccharomyces cerevisiae
Source: mSphere. 2015 Dec 16;1(1):e00038-15. doi: 10.1128/mSphere.00038-15 (PMC4863634; doi:10.1128/mSphere.00038-15)
Supplement: Table S1 [file sph001160039st1.pdf]

Table S1. Genes screened in this study

gene name	systematic name						
ACS1	YAL054C	*CDC40*	YDR364C	*GCN5*	YGR252W	*MND2*	YIR025W
ADA2	YDR448W	*CKB1*	YGL019W	*GCS1*	YDL226C	*MRK1*	YDL079C
ADH7	YCR105W	*CLB5*	YPR120C	*GEA1*	YJR031C	*MTC6*	YHR151C
AGE2	YIL044C	*CLU1*	YMR012W	*GEP5*	YLR091W	*MUM2*	YBR057C
AIM36	YMR157C	*COA1*	YIL157C	*GET1*	YGL020C	*MUM3*	YOR298W
AKR1	YDR264C	*CRS5*	YOR031W	*GIM5*	YML094W	*MUS81*	YDR386W
AMA1	YGR225W	*CSF1*	YLR087C	*GON7*	YJL184W	*NAT1*	YDL040C
APL3	YBL037W	*CST9*	YLR394W	*GPB2*	YAL056W	*NBP2*	YDR162C
APS3	YJL024C	*CTK1*	YKL139W	*GRX3*	YDR098C	*NEM1*	YHR004C
APT1	YML022W	*CTK3*	YML112W	*GSP2*	YOR185C	*NGG1*	YDR176W
ARC1	YGL105W	*CTS2*	YDR371W	*HMG2*	YLR450W	*NMD2*	YHR077C
ARC18	YLR370C	*CYC8*	YBR112C	*HMT1*	YBR034C	*NPR3*	YHL023C
ARD1	YHR013C	*DEF1*	YKL054C	*HOP2*	YGL033W	*NUP120*	YKL057C
ARG82	YDR173C	*DEP1*	YAL013W	*HPR1*	YDR138W	*NUP170*	YBL079W
ARO2	YGL148W	*DHH1*	YDL160C	*HSL7*	YBR133C	*NUP84*	YDL116W
ASE1	YOR058C	*DIA2*	YOR080W	*HTD2*	YHR067W	*NUR1*	YDL089W
ATG1	YGL180W	*DID4*	YKL002W	*ICT1*	YLR099C	*PAU8*	YAL068C
ATG1	YPR049C	*DMC1*	YER179W	*IDS2*	YJL146W	*PBP1*	YGR178C
ATG10	YLL042C	*DOA1*	YKL213C	*IFA38*	YBR159W	*PCK1*	YKR097W
ATG12	YBR217W	*DRS2*	YAL026C	*IGO2*	YHR132W-A	*PEX12*	YMR026C
ATG13	YPR185W	*EAF1*	YDR359C	*IMP2'*	YIL154C	*PEX25*	YPL112C
ATG15	YCR068W	*EAF6*	YJR082C	*IRA2*	YOL081W	*PEX31*	YGR004W
ATG16	YMR159C	*ECM8*	YBR076W	*ISC1*	YER019W	*PFK2*	YMR205C
ATG18	YFR021W	*EMI1*	YDR512C	*ISC10*	YER180C	*PFK26*	YIL107C
ATG2	YNL242W	*EMI2*	YDR516C	*KAP120*	YPL125W	*PGD1*	YGL025C
ATG29	YPL166W	*EMP70*	YLR083C	*KCS1*	YDR017C	*PKH2*	YOL100W
ATG3	YNR007C	*ENT4*	YLL038C	*LAS21*	YJL062W	*PLB1*	YMR008C
ATG31	YDR022C	*EPS1*	YIL005W	*LEO1*	YOR123C	*PLB2*	YMR006C
ATG5	YPL149W	*ERG4*	YGL012W	*LGE1*	YPL055C	*POG1*	YIL122W
ATG7	YHR171W	*ERV14*	YGL054C	*LRS4*	YDR439W	*POP2*	YNR052C
ATG8	YBL078C	*ERV25*	YML012W	*MAD3*	YJL013C	*PPH3*	YDR075W
ATG9	YDL149W	*FAB1*	YFR019W	*MAF1*	YDR005C	*PRB1*	YEL060C
BAR1	YIL015W	*FAR11*	YNL127W	*MAM1*	YER106W	*PRS3*	YHL011C
BLI1	YKL061W	*FAT1*	YBR041W	*MCK1*	YNL307C	*PTC1*	YDL006W
BPH1	YCR032W	*FBP1*	YLR377C	*MEI5*	YPL121C	*PTC5*	YOR090C
BSD2	YBR290W	*FIS1*	YIL065C	*MET1*	YKR069W	*RAD33*	YML011C
BST1	YFL025C	*FMP46*	YKR049C	*MET13*	YGL125W	*RAD51*	YER095W
BTS1	YPL069C	*FMS1*	YMR020W	*MET22*	YOL064C	*RAD55*	YDR076W
BUD22	YMR014W	*FUN12*	YAL035W	*MLS1*	YNL117W	*RAD57*	YDR004W
CBC2	YPL178W	*GAL11*	YOL051W	*MMS4*	YBR098W	*RAD6*	YGL058W

gene name	systematic name				
RAI1	YGL246C	*SHP1*	YBL058W	*THR4*	YCR053W
RAM1	YDL090C	*SIN3*	YOL004W	*TIF4631*	YGR162W
RCY1	YJL204C	*SKN1*	YGR143W	*TIR1*	YER011W
RDH54	YBR073W	*SLM3*	YDL033C	*TMA64*	YDR117C
REC8	YPR007C	*SLX5*	YDL013W	*TOM1*	YDR457W
REF2	YDR195W	*SLX8*	YER116C	*TPS1*	YBR126C
RIM101	YHL027W	*SLZ1*	YNL196C	*TPS2*	YDR074W
RIM11	YMR139W	*SMA2*	YML066C	*TRM9*	YML014W
RIM15	YFL033C	*SMK1*	YPR054W	*TUL1*	YKL034W
RIM4	YHL024W	*SNF2*	YOR290C	*TUP1*	YCR084C
RMD1	YDL001W	*SNF3*	YDL194W	*UBP14*	YBR058C
RMD5	YDR255C	*SNF4*	YGL115W	*UFD2*	YDL190C
RMD6	YEL072W	*SNF6*	YHL025W	*UME1*	YPL139C
RMD7	YER083C	*SNF7*	YLR025W	*VID28*	YIL017C
RMD8	YFR048W	*SPF1*	YEL031W	*VMS1*	YDR049W
RMD9	YGL107C	*SPO1*	YNL012W	*VPH1*	YOR270C
RPA49	YNL248C	*SPO16*	YHR153C	*VPS13*	YLL040C
RPL14A	YKL006W	*SPO19*	YPL130W	*VPS20*	YMR077C
RPL19A	YBR084C-A	*SPO20*	YMR017W	*VPS24*	YKL041W
RPL27A	YHR010W	*SPO71*	YDR104C	*VPS27*	YNR006W
RPL2A	YFR031C-A	*SPO73*	YER046W	*VPS3*	YDR495C
RPL2B	YIL018W	*SPO75*	YLL005C	*VPS4*	YPR173C
RPL31A	YDL075W	*SPO77*	YLR341W	*VPS52*	YDR484W
RPL34B	YIL052C	*SPR3*	YGR059W	*VPS53*	YJL029C
RPL40A	YIL148W	*SPS1*	YDR523C	*VPS54*	YDR027C
RPL7A	YGL076C	*SPT10*	YJL127C	*WHI3*	YNL197C
RTC6	YPL183W-A	*SPT20*	YOL148C	*YAP1*	YML007W
RTF1	YGL244W	*SPT3*	YDR392W	*YET3*	YDL072C
SAC7	YDR389W	*SPT4*	YGR063C	*YPF1*	YKL100C
SAE2	YGL175C	*SRT1*	YMR101C	*YSP2*	YDR326C
SAE3	YHR079C-A	*SRV2*	YNL138W	*YVH1*	YIR026C
SAP190	YKR028W	*SSF1*	YHR066W	*ZIP1*	YDR285W
SCJ1	YMR214W	*SSN8*	YNL025C	*ZUO1*	YGR285C
SCO1	YBR037C	*SSO2*	YMR183C		YBR090C
SCS2	YER120W	*SSP2*	YOR242C		YDL119C
SDH2	YLL041C	*SSZ1*	YHR064C		YEL020C
SDH5	YOL071W	*STP4*	YDL048C		YGR111W
SDH7	YDR511W	*SWF1*	YDR126W		YHR202W
SDS3	YIL084C	*SWF5*	YOR333C		YHR210C
SEC22	YLR268W	*SWI3*	YJL176C		YJL160C
SED4	YCR067C	*SWM1*	YDR260C		YJR079W
SFK1	YKL051W	*SWS2*	YNL081C		YNL146W
SGF29	YCL010C	*TGL4*	YKR089C		YOR008C-A
SGF73	YGL066W	*TGS1*	YPL157W		
SHE9	YDR393W	*THP2*	YHR167W		
