# Supplementary material for: The Dysferlin Domain-Only Protein, Spo73, Is Required for Prospore Membrane Extension in Saccharomyces cerevisiae
Source: mSphere. 2015 Dec 16;1(1):e00038-15. doi: 10.1128/mSphere.00038-15 (PMC4863634; doi:10.1128/mSphere.00038-15)
Supplement: Table S2 [file sph001160039st2.pdf]

Table S2. Construction of strains

Yesat Strains

Locus	Construction
spo71Δ::kanMX6	HT403 and HT404, pFA6a-kanMX6 (1)
spo73Δ::kanMX6	IC7 and IC8, pFA6a-kanMX6 (1)
ura3::URA3-DON1-GFP	306-DON1-GFP
spo1Δ::kanMX6	HT372 and HT373, genomic DNA of *spo1Δ::kanMX6* strain form deletion collection
spo1Δ::HIS3MX6	pFA6a-His3MX6 (1) (*spo1Δ::kanMX6* to *spo1Δ::HIS3MX6*)
spo73Δ::HIS3MX6	pFA6a-His3MX6(1) (*spo73Δ::kanMX6* to *spo73Δ:HIS3MX6*)
ssp1Δ*::kanMX6*	HT86 and HT87, pFA6a-kanMX6 (1)
VPS13::GFP-HIS3MX6	IC4 and IC5, pFA6a-yEGFP-HIS3MX6 (2)
HIS3	TN377 and TN378, pRS303 (3) (*his3ΔSK* to *HIS3*)

1. **Longtine MS, Iii AMK, Demarini DJ, Shah NG**. 1998. Additional Modules for Versatile and Economical PCR-based Gene Deletion and Modification in *Saccharomyces cerevisiae*. Yeast **961**:953–961.

2. **Nickas ME, Neiman AM**. 2002. Ady3p Links Spindle Pole Body Function to Spore Wall Synthesis in *Saccharomyces cerevisiae*. Genetics **1450**:1439–1450.

3. **Sikorski RS, Hieter P**. 1989. A System of Shuttle Vectors and Yeast Host Strains Designed for Efficient Manipulation of DNA in. Genetics **122**:19–27.
