# Supplementary material for: The Dysferlin Domain-Only Protein, Spo73, Is Required for Prospore Membrane Extension in Saccharomyces cerevisiae
Source: mSphere. 2015 Dec 16;1(1):e00038-15. doi: 10.1128/mSphere.00038-15 (PMC4863634; doi:10.1128/mSphere.00038-15)
Supplement: Table S3 [file sph001160039st3.pdf]

Table S3. Primers used in this study

Name	Sequence (5’→3’)
HT403	TTCGAAGGATCTCTTTTCTTC
HT404	TGAGGAGGAAGGTACTAATG
IC7	GAATCTTGTCTACGTAATTG
IC8	TGTTCTGCATAATGTCACCT
HT372	AAAACATACTTCAGCGGCTA
HT373	ATGGCAAGATTTTATTACTG
HT86	ACAATAGTGCCTATTATCATGATAGAAGTAGAGTAGAAAA GCTAGCAACACGGATCCCCGGGTTAATTAA
HT87	CAATAAGCATAGAACATGGAATGAGTGTTCAAACTATATT CGTTTGTTTTGAATTCGAGCTCGTTTAAAC
IC4	ACATCGCCATTGCTGTTAGAGAATACAATAAGTACTGTGAA GCTATCCTACGGATCCCCGGGTTAATTAA
IC5	TAATACGATTGAATTATAGCTACATAGTGTACAAAAGCGGG TATATACTTGAATTCGAGCTCGTTTAAAC
OK13	AAACCTTATTATATGCCTTTACATATACTGTCACGCGACATT TTCACCTTGAATTCGAGCTCGTTTAAAC
OK14	ACGTCCTCCGGTAAAGCAGAGAAATCCTTCAAGAAATGGTT TTTGCCCATTTTGTATAGTTCATCCATGC
HT369	GAGAAGGTACCTAAGTCTAGGCGCTTTCAA
OK15	GAAGAATTCCACTACGAGATCTGGGTGAT
OK29	TGGAATTTCAATAACTCTGCTTGGGCTAGCGCTAATGGCTTG GTGAG
OK30	CTCACCAAGCCATTAGCGCTAGCCCAAGCAGAGTTATTGAAATTCCA
HT421	GAAGAAGGTACCCTTGCTTCGATTTCGGTAGT
HT422	GAAGAATTAATTAAAGACATATATCTCTATGTATGAT
OK11	GAAGAACCGCGGAAATGATGATGAAAGGACTG
HT424	GAAGAATTCGGGTGAACATAGCAATATAG
TN194	GAAGAAGAAGCGGCCGCAACATCCAAAAGGAATCTACTC
TN197	GAAGAAGGTACCACTTGGCTCGTCTGGATTCC
TN62	GAAGAACTCGAGATTATATATTAAATTTGCTC
HT66E	GAAGAATTCAGATCTATATTACCCTGTTATCC
TN377	CGTTTTAAGAGCTTGGTGAG
TN378	TCGAGTTCAAGAGAAAAAAAAAG
