# Supplementary material for: The Dysferlin Domain-Only Protein, Spo73, Is Required for Prospore Membrane Extension in Saccharomyces cerevisiae
Source: mSphere. 2015 Dec 16;1(1):e00038-15. doi: 10.1128/mSphere.00038-15 (PMC4863634; doi:10.1128/mSphere.00038-15)
Supplement: Table S4 [file sph001160039st4.pdf]

Table S4. Plasmids used in this study

Name	Protein expressed	Source
pRS424-G20	GFP- $Spo20^{51-91}$	(1)
pRS426-R20	mRFP-$Spo20^{51-91}$	(2)
306-DON1-GFP	Don1-GFP	This study
316-HTB2-mCherry	Htb2-mCherry	This study
pRS424-*DTR1-RFP*	Dtr1-mRFP	(3)
pRS426-P_{PRC1} -GFP-PH^{OSH2}	GFP-PH^{OSH2}	(4)
424- P_{SPO20}-GFP-SPO73	GFP-Spo73	This study
424- P_{SPO20}-GFP-SPO73-AAA	GFP-Spo73 $^{R119A\ K121A\ K123A}$	This study
314- P_{SPO73}-GFP-SPO73	GFP-Spo73	This study
314- P_{SPO73}-GFP-SPO73-AAA	GFP-Spo73 $^{R119A\ K121A\ K123A}$	This study
424-SPO71-GFP	Spo71-GFP	(5)
426-SPO71	Spo71	This study
426-VPS13	Vps13	This study

1. **Nakanishi H, de los Santos P, M. NA**. 2004. Positive and Negative Regulation of a SNARE Protein by Control of Intracellular Localization. Mol Biol Cell **15**:1802–1815.
2. **Suda Y, Nakanishi H, Mathieson EM, Neiman AM**. 2007. Alternative modes of organellar segregation during sporulation in *Saccharomyces cerevisiae*. Eukaryot Cell **6**:2009–17.
3. **Park J-S, Neiman AM**. 2012. *VPS13* regulates membrane morphogenesis during sporulation in *Saccharomyces cerevisiae*. J Cell Sci **125**:3004–3011.
4. **Yu JW, Mendrola JM, Audhya A, Singh S, Keleti D, DeWald DB, Murray D, Emr SD, Lemmon M a.** 2004. Genome-wide analysis of membrane targeting by *S. cerevisiae* pleckstrin homology domains. Mol Cell **13**:677–688.
5. **Park JS, Okumura Y, Tachikawa H, Neiman AM**. 2013. *SPO71* encodes a developmental stage-specific partner for Vps13 in *Saccharomyces cerevisiae*. Eukaryot Cell **12**:1530–1537.
